# Fusion of the Greater and Suboccipital Nerves: A Case Report With Application to Patients With Occipital Neuralgia

**DOI:** 10.7759/cureus.24815

**Published:** 2022-05-07

**Authors:** Rex Wang, Joe Iwanaga, Łukasz Olewnik, Aaron S Dumont, R. Shane Tubbs

**Affiliations:** 1 Structural & Cellular Biology, Tulane University School of Medicine, New Orleans, USA; 2 Neurosurgery, Tulane University School of Medicine, New Orleans, USA; 3 Anatomical Dissection and Donation, Medical University of Lodz, Lodz, POL; 4 Neurosurgery and Ochsner Neuroscience Institute, Ochsner Health System, New Orleans, USA

**Keywords:** variations, suboccipital nerve, greater occipital nerve, cadaver, anatomy

## Abstract

Atypical presentations of occipital neuralgia might have an anatomical cause. Therefore, a better understanding of variant anatomy in this region can help physicians who treat such patients. During the dissection of the suboccipital region in an 83-year-old at-death male cadaver, an unusual finding was noted between the suboccipital and greater occipital nerves. No branches from this segment of the suboccipital nerve were identified. Therefore, initially, the suboccipital muscles were thought to be innervated not by the suboccipital nerve but rather by branches of the medial (greater occipital nerve) and lateral branches of the C2 dorsal ramus. However, with microdissection, these fibers were found to ascend with the medial branch of the C2 ramus (greater occipital nerve) and to distribute fibers to the rectus capitis minor and major and then continue with the greater occipital nerve to the skin over the occiput. No fibers from the suboccipital nerve traveled to the C2 spinal nerve or its lateral branch. The lateral part of the dorsal ramus of C2 innervated the obliquus capitis superior and obliquus capitis inferior. Additionally, a long slender branch from the lateral branch of the C2 dorsal ramus traveled medially to innervate the skin over the C2 spinous process. This case demonstrates that some fibers in the greater occipital nerve (C2), both cutaneous and motor, can be derived from the suboccipital nerve (C1). This information can help in diagnosing some patients with atypical presentations and can help better target all involved occipital nerves.

## Introduction

Since its first description by Galen (ca. 173 CE), the first cervical spinal nerve has been noted to have variability in its presence, the number of rootlets it contains, the presence or absence of a dorsal root ganglion, anastomotic connections with other nerves in this region, and the names given to the C1 nerve root on the whole as well as its branches [1,2]. For clarity and consistency with the most recent nomenclature, here, the entire nerve will be referred to as the C1 spinal nerve, and the dorsal ramus of this nerve will be referred to as the suboccipital nerve.

The dorsal ramus of the C1 spinal nerve, the suboccipital nerve, is located within the suboccipital triangle between the occipital bone and atlas, with the vertebral artery bordering the nerve superiorly where it enters the foramen magnum, and the posterior arch of the atlas bordering the nerve inferiorly [1]. Although it primarily serves to provide motor innervation to the short suboccipital muscles, the rectus capitis posterior minor, and the semispinalis capitis, it may give off a cutaneous branch supplying the skin overlying the occiput, as reported by Lake et al. [3]. The sensory innervation of this variant branching is not very well understood [4], but it may be a cause of recalcitrant occipital neuralgia. Additionally, knowledge of other anatomical variations of the suboccipital nerve is important to clinicians who treat patients with occipital neuralgia to better understand presenting signs and symptoms. The present cadaveric case reports an unusual finding of the suboccipital nerve that should be borne in mind by physicians.

## Case presentation

**Figure 1:**
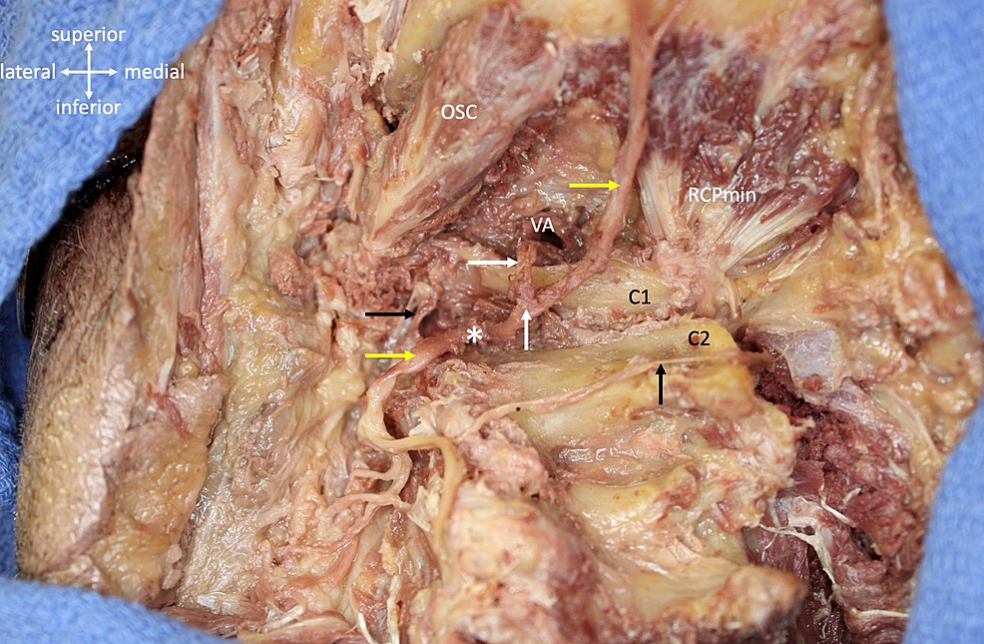
Cadaveric dissection over the left suboccipital region. Note the obliquus superior capitis (OSC), left and right rectus capitis minor muscles (RCPmin), posterior arch of C1 (C1), spinous process of C2 (C2), and transected vertebral artery (VA). The obliquus inferior capitis and rectus capitis major have been removed. All of the suboccipital muscles were innervated not by the suboccipital nerve but rather by branches of the medial and lateral branches of the C2 dorsal ramus (e.g., horizontal black arrow). The C2 dorsal ramus (*) is seen splitting into its lateral branches (left yellow arrow) and medial branch (right yellow arrow), also known as the greater occipital nerve. Note the suboccipital nerve (upper white arrow) emerging inferiorly to the vertebral artery and just above the posterior arch of C1 to then fuse with the medial branch of the C2 dorsal ramus at the lower white arrow. Lastly, note the long slender branch (black arrow) from the lateral branch of the C2 dorsal ramus which traveled medially to innervate the skin over the C2 spinous process.

**Figure 2:**
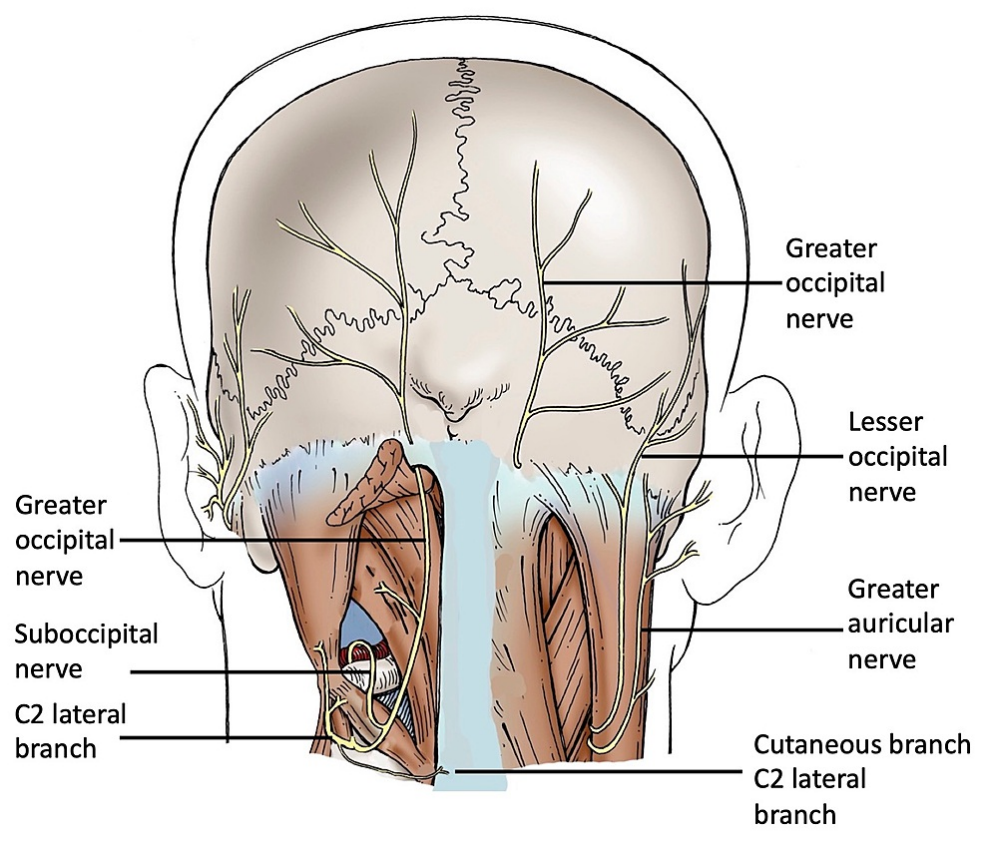
Schematic drawing of the case described here and shown in Figure 1. Note the suboccipital nerve on the left side emerges between the vertebral artery and posterior arch of C1 and then soon after fuses with the medial branch, that is, greater occipital nerve of the C2 spinal nerve. Note that the left greater occipital nerve supplying the rectus capitis major and the C2 lateral branch giving rise to branches supplying the obliquus capitis superior and obliquus capitis inferior and a cutaneous branch over the C2 spinous process in the midline.

During the dissection of the suboccipital region in an 83-year-old at-death male cadaver (fresh frozen), an unusual finding was noted between the suboccipital and greater occipital nerves. The suboccipital nerve was observed to be emerging from the inferior to the vertebral artery and just above the posterior arch of C1 to then travel inferior to the arch for a distance of 1.2 cm and then fuse with the medial branch of the C2 dorsal ramus (Figures 1, 2).

No branches from the suboccipital nerve were identified. Therefore, initially, the suboccipital muscles were thought to be innervated not by the suboccipital nerve but rather by branches of the medial (greater occipital nerve) and lateral branches of the C2 dorsal ramus. However, with microdissection, these fibers were found to ascend with the medial branch of the C2 ramus (greater occipital nerve) and to distribute fibers to the rectus capitis minor and major and then continue with the greater occipital nerve to the skin over the occiput. No branches from the suboccipital nerve traveled to the C2 spinal nerve or its lateral branch. The lateral part of the dorsal ramus of C2 innervated the obliquus capitis superior and obliquus capitis inferior. Additionally, a long slender branch from the lateral branch of the C2 dorsal ramus traveled medially to innervate the skin over the C2 spinous process (Figures 1, 2). No similar variations in the suboccipital nerve and greater occipital nerve were observed on the contralateral side of the specimen.

## Discussion

The C1 spinal nerve has been noted to have anastomotic connections with both the spinal accessory nerve and hypoglossal nerve [3]. However, none of these connections is thought to provide cutaneous innervation to the surrounding region. Interestingly, the present case demonstrates the dorsal ramus of C1, the suboccipital nerve [4], using the greater occipital nerve to disseminate many of its branches, both cutaneous and motor.

Fusion of the greater and suboccipital nerves, as seen in the present case, may be a potential consideration in patients with occipital neuralgia who have failed successive treatments, including a diagnostic anesthetic nerve blockade of the nerves innervating the occipital scalp. Interestingly, a dorsal root ganglion of the C1 spinal nerve only about 10% of the time [3]. During development, sensory neurons of the suboccipital nerve course are found coursing along the spinal accessory nerve. Lake et al. [3] classified the variation in the C1 spinal nerve into at least four subtypes. In type I, the dorsal roots of the C1 spinal nerve are absent, and it joins with the spinal accessory nerve. In type II, ventral and dorsal roots are both present, and it does not connect with any other nerves in the region. In type III, the C1 spinal nerve connects with the spinal accessory nerve via Mackenzie’s nerve. In type IV, the dorsal roots are absent, and there is a connection with the spinal accessory nerve as well as the ventral roots of the C1 spinal nerve via Mackenzie’s nerve.

Occipital neuralgia is typically caused by entrapment of the greater occipital nerve, with some cases arising from compression of other occipital nerves. The greater occipital nerve is composed of fibers from the dorsal ramus of the C2 spinal nerve. It travels between the C1 and C2 vertebrae, passing between the inferior capitis oblique and semispinalis capitis muscles, and continues by piercing the semispinalis muscle to then be distributed to the skin on the posterior scalp [5]. Due to its close relationship with the nearby muscles, the greater occipital nerve may become compressed in these regions.

## Conclusions

A deeper knowledge of the variations of the suboccipital nerve can help clinicians who treat patients with occipital neuralgia. The current case demonstrates that some fibers in the greater occipital nerve (C2), both cutaneous and motor, can be derived from the suboccipital nerve (C1). Such information can help in diagnosing patients with atypical presentations and can help better target all involved occipital nerves.
